# Health Behavior Theory in Physical Activity Game Apps: A Content Analysis

**DOI:** 10.2196/games.4187

**Published:** 2015-07-13

**Authors:** Hannah E Payne, Victor BA Moxley, Elizabeth MacDonald

**Affiliations:** ^1^ Computational Health Science Research Group Department of Health Science Brigham Young University Provo, UT United States; ^2^ Human Performance Research Group Department of Exercise Science Brigham Young University Provo, UT United States

**Keywords:** health and fitness apps, mobile phone, behavioral health, theory, content analysis, physical activity

## Abstract

**Background:**

Physical activity games developed for a mobile phone platform are becoming increasingly popular, yet little is known about their content or inclusion of health behavior theory (HBT).

**Objective:**

The objective of our study was to quantify elements of HBT in physical activity games developed for mobile phones and to assess the relationship between theoretical constructs and various app features.

**Methods:**

We conducted an analysis of exercise and physical activity game apps in the Apple App Store in the fall of 2014. A total of 52 apps were identified and rated for inclusion of health behavior theoretical constructs using an established theory-based rubric. Each app was coded for 100 theoretical items, containing 5 questions for 20 different constructs. Possible total theory scores ranged from 0 to 100. Descriptive statistics and Spearman correlations were used to describe the HBT score and association with selected app features, respectively.

**Results:**

The average HBT score in the sample was 14.98 out of 100. One outlier, SuperBetter, scored higher than the other apps with a score of 76. Goal setting, self-monitoring, and self-reward were the most-reported constructs found in the sample. There was no association between either app price and theory score (*P*=.5074), or number of gamification elements and theory score (*P*=.5010). However, Superbetter, with the highest HBT score, was also the most expensive app.

**Conclusions:**

There are few content analyses of serious games for health, but a comparison between these findings and previous content analyses of non-game health apps indicates that physical activity mobile phone games demonstrate higher levels of behavior theory. The most common theoretical constructs found in this sample are known to be efficacious elements in physical activity interventions. It is unclear, however, whether app designers consciously design physical activity mobile phone games with specific constructs in mind; it may be that games lend themselves well to inclusion of theory and any constructs found in significant levels are coincidental. Health games developed for mobile phones could be potentially used in health interventions, but collaboration between app designers and behavioral specialists is crucial. Additionally, further research is needed to better characterize mobile phone health games and the relative importance of educational elements versus gamification elements in long-term behavior change.

## Introduction

Serious games, or games whose primary purpose is to educate rather than entertain [1], have become a popular research focus because of their potential application in fields such as education, military, business, and health and wellness [2]. Games appear to be an emerging option for behavioral change, especially health behaviors, as serious games address innate psychological needs while offering intrinsic motivation in the form of fun [3]. Serious games may also have potential to impact health behavior change on a widespread level because of their appeal and the popularity of gaming. Furthermore, 59% of Americans play video games, and the average household owns at least one game console, PC, or mobile phone [4]. Serious games have been increasingly utilized in public health interventions [5], and many have shown promise in changing behavior in areas such as tobacco cessation, violence prevention, and mental health [6-8].

Serious games for public health have typically been developed as video games [9,10]. Exergames, or video games that require physical movement in order to play, are a particularly popular tool for health professionals especially as physical activity among the US population has decreased and chronic diseases such as obesity have increased [11-13]. While serious games were initially designed for personal computers and more recently for gaming systems, mobile phones are another increasingly viable platform for health and physical fitness games for several reasons. First, mobile phones are widely used; mobile phone use increased 22% over the year 2013 alone, and among US households that own a device to play video games, 53% play games on a mobile phone [4]. Second, game-like elements are already frequently utilized by health app developers. Lister et al found that elements of gamification, or the “use of game design elements in non-game contexts” [14], appeared in a large percentage of health and fitness apps [15]. Third, the use of apps in health interventions is already prevalent among public health professionals [16-18], and interventions using serious health games on mobile phones are emerging as well [19-21]. The few health behavior change interventions that have utilized serious games developed for a mobile phone platform have shown to improve health outcomes in areas such as diabetes management and healthy eating [19-21].

Much research suggests that health interventions designed around health behavior theory (HBT) are more effective in changing behavior than those which are not [22-25]. Many content analyses of health apps indicate that such apps generally contain low levels of HBT or are not adequately designed for long-term behavior change [26-31]. For example, Breton et al [32] noted in a content analysis of weight-loss apps that most contained few evidence-based practices, and Cowan et al [28] reported in a content analysis of physical activity apps that the sample contained low levels of HBT, suggesting that the lack of behavioral components may have been due to the widely varying professional backgrounds of app developers. Conversely, in a content analysis of physical activity video games, Lyons et al [33] reported that the games contained a relatively high percentage of health behavior constructs [33]. It may be that serious health games in general contain high amounts of HBT, whether HBT is consciously included or because games by design are more conducive to inclusion of HBT, though there has not been enough research conducted to determine whether this is true.

As health professionals are increasingly using mobile phone apps in interventions to increase physical activity, research on the content of such apps is important. Although many content analyses for health and fitness apps have been recently conducted [26-31] and analyses of exergames are emerging [33], currently, no studies have been conducted to investigate HBT in serious games developed for mobile phones. The purpose of our study was to identify the currently available most popular physical activity health games developed for mobile phones and to conduct a content analysis of HBT in these games.

## Methods

### Study Design

Our study was a content analysis of HBT contained in physical activity game apps selected from among the apps available in the iTunes App Store’s Health and Fitness category. Two graduate students trained in HBT coded the apps.

### Sample Identification

The sample was collected from the Apple App Store in the fall of 2014. Apps designed for iPhone use were chosen, because many similar app content analyses have used Apple’s App store for sample selection [27,28,30,31]. This sample contained apps categorized under the health and fitness section of the App Store and were related to physical activity. Physical activity mobile phone games were selected for two reasons: (1) physical activity is both an impactful and neglected health behavior [12,13], and (2) interventions utilizing physical activity apps are a growing area of interest for health researchers [34-37]. There were 42 key search terms that were established prior to the sample collection, using key phrases for both physical activity and games. Keywords included fitness terms such as “running”, “walking”, “workout”, “exercise”, and others related to these behaviors, as well as game keywords, including “challenge”, “adventure”, and “interactive” (Table 1).

**Table 1:** Search terms.

Physical activity terms	Gamification terms
Dance	Game
Exercise	Avatar
Fitness	Reality game
Run	Virtual
Fit	Challenge
Team	Race
Train	Quest
Trainer	Adventure
Goal	Interactive
Walk	Simulator
Track	Augmented reality
Tracker	Running
Trek	Workout
Health	Cycling
Aerobics	Cardio
Weight training	

We have formal training in public health and health behavior and adapted the search terms from a previous content analysis of health theory in fitness apps [28]. Search terms were entered into the Apple App Store on iPads, as iPads allow filtering of results. Search results were narrowed by (1) iPhone only, (2) health and fitness, and (3) popularity. Previous content analyses of apps ordered search results by popularity to ensure that the apps that were reviewed were highly used [15,28].

The first 500 most popular apps were chosen for each search term, as the app store does not sort by page number. Additionally, as adapted from Lister et al [15], searching through a set number of primary results is enough for an adequate sample, because users do not typically search beyond the first few search pages [38,39].

The detailed written descriptions for the first 500 apps that appeared in the search results under each topic were analyzed to assess whether each app met the criteria for a serious health game. The definition for serious game was taken from definitions provided by Michael and Chen [1] and Shegog [19] and required that the app was primarily intended to educate, rather than entertain, as well as change a health behavior; additionally, each app needed to either (1) contain a fantasy storyline or narrative, or (2) include the possibility of failure.

The initial search revealed 86 apps that were originally selected as meeting the criteria. After reviewing all of the apps, 52 were identified for final inclusion. Apps were excluded that required special equipment (e.g., bikes, treadmills, pedometers, heart rate meters, GPS) (8), could not be located in the App Store upon subsequent searches (5), failed to operate (8), or upon further investigation did not meet the criteria for a physical activity game (13).

### Coding Procedure

Each app selected for final inclusion was coded into an initial sampling rubric using Qualtrics online survey software. The coders downloaded each app to an iPad or iPhone and played each game for a minimum of 30 minutes or until completing one level to increase familiarity with the user interface and available functions. The coders then used a theory-based instrument to conduct the content analysis for each app.

### Measurement

The instrument and methodology used for coding was adapted from an instrument used by Cowan et al [28] designed to assess theoretical content of physical activity apps. A similar rubric was used, with the addition of more questions about social networking sites utilized in the apps, expanding the options for type of exercise utilized in the app, and tailoring the items for a serious game setting.

Each app was coded using a rubric with 100 theoretical items, containing five questions each for 20 different constructs, as used by Cowan et al [28]. The coding rubric required choosing 0 points when the construct was not present in the app, 1 point for generalized information on the construct, 2 points for assessing user’s knowledge relating to the construct, 3 points for providing feedback about user’s knowledge on the construct, 4 points for general assistance relating to the construct, and 5 points for individualized assistance to improve relative to the construct. The points for each construct were added together for each app. This resulted in a HBT score ranging from 0 to 100.

We also coded for gamification elements. The specific gamification elements coded for were selected from an instrument used by Lister et al [15] and from the definition adapted from Michael and Chen [1] and Shegog [19] (Table 6).

### Analysis

Coded data were imported from Qualtrics and analyzed using SAS Studio. To verify the level of interrater reliability, both coders independently coded 10 common apps, approximately 12% (10/86) of the original sample and 19% (10/52) of those retained for final inclusion. A Cohen’s kappa coefficient, a method commonly used in content analysis research, was calculated to measure interrater agreement (*κ*=.60) with 97% agreement [15,28]. This is categorized as high-moderate agreement, which ranges from .41 to.60 and is an acceptable level of interrater agreement [40]. Descriptive statistics were used to report on the integration of HBT into physical activity mobile phone games. A Spearman correlation was used to determine whether there was an association between HBT and price, as well as HBT and total number of gamification elements.

## Results

### Sample Characteristics

Characteristics of the sample are shown in Table 2.The mean HBT score was 14.98 out of 100 points. One of the apps, SuperBetter, was an outlier with a much higher theory score than the rest of the sample (HBT score of 76 out of 100). Four of the apps (GPS Invaders, MotionMaze Holiday Adventure, Mapventures, and TrezrHunt Free) contained no HBT elements as dictated by the coding rubric. Walking (56%, 29/50) and running (42%, 22/50) were the most common exercises incorporated into apps (Table 3).

**Table 2:** App characteristics.

App name	HBT score	App name	HBT score
SuperBetter	76	Rare Candy—Epic Habit and Goa	12
Yoga Retreat	37	Rare candy free	12
Zombies, Run! 5k Training	35	Wokamon	12
iBelly Workout	29	Silk Road Walk	12
The Walk	29	Runno	11
Workout in a Bag—for kids	29	Box the Bag	9
Yes, Drill Sergeant!	28	Block Sports	9
RunAlice	28	Walky	8
Walk it!	24	AR Basketball	8
Zombies Run!	24	Battlesuit Runner Fitness	8
Ninja Fitness Free	24	Jump Boy	8
Streetquest—run a game	23	iBowl	8
Walk n' Play	22	MotionMaze Trick or Treat	7
Burn Your Fat with Me!	21	Hike the World—GPS Tracker	6
PushUp Club Free	20	MotionMaze	6
NFL Play 60	19	Paranoid	5
Habit Monster	17	treasure island GPS,	5
Turfly	17	AR Soccer	4
Daily Spartan	17	Keep Moving	4
Walkr—Galaxy Adventure in You	16	Pygmalions Challenge	1
FitQuest Lite	16	GPS Fun Lite	1
RunZombieRun	16	Gigaputt	1
7 Min Workout Zombie Survival	15	GPS Invaders	0
HuntedApp	14	MotionMaze Holiday Adventure	0
TapCloud	13	Mapventures	0
Superhero Workout	13	TrezrHunt Free	0

**Table 3:** Exercise type in apps.

Forms of exercise	n	%
Walking	29	56
Running	22	42
None/general movement	17	33
Weight lifting/bodyweight exercises	13	25
Other	9	17
Stretching	8	15
Jumping	5	10

### Presence of Specific HBT Constructs

Table 4 shows the mean score (0-5) for the presence of HBT constructs measured in the sample, as well as the overall score (0-100). Goal setting, self-monitoring, and self-reward were the most-reported constructs found. Only self-monitoring and goal-setting had a median score greater than zero. For both, the median score was 5.

**Table 4:** HBT score by construct (N=52).

HBT		n (%)	Median	Mean	SD
**Overall score**		52 (100)	12.5	14.98	12.92
**Capacity**					
	General information	20 (38)	0	1.48	1.99
	Self-monitoring	30 (58)	4	2.25	1.99
	Stress management	3 (6)	0	0.23	0.94
	Time management	5 (10)	0	0.33	1.08
	Learning	4 (8)	0	0.27	1.03
**Motivation**					
	Incentives	13 (25)	0	0.65	1.37
	Barriers	7 (13)	0	0.44	1.26
	Risks	10 (19)	0	0.62	1.39
	Goal-setting	36 (69)	4	2.25	1.87
	Self-reward	23 (44)	0	1.77	2.01
	Readiness	9 (17)	0	0.5	1.18
	Self-talk	4 (8)	0	0.31	1.08
	Self-efficacy	9 (17)	0	0.63	1.46
	Norms	3 (6)	0	0.17	0.79
**Opportunity/trigger**					
	Peer pressure	18 (35)	0	1.33	1.89
	Modeling	16 (31)	0	0.83	1.50
	Relapse prevention	2 (4)	0	0.10	0.57
	Follow-up	6 (12)	0	0.38	1.09
	Guilt	4 (8)	0	0.19	0.79
	Stimulus control	4 (8)	0	0.25	0.95

### Price and HBT

**Figure 1:**
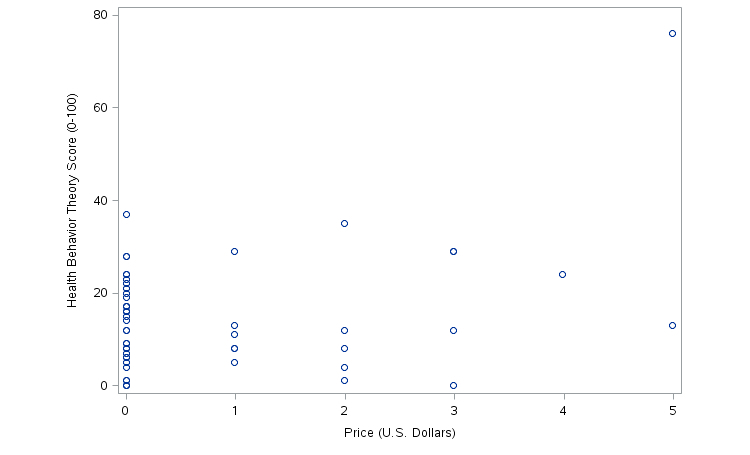
Price and HBT score.

There was no association between price and HBT (Spearman correlation coefficient *R*_S_=0.09641, *P*=.5010) (Figure 1). However, SuperBetter, which had the highest HBT score, also had the highest price (Figure 1, Table 5).

**Table 5:** Physical activity games by price.

Price (US$)	Apps
0	34
0.99	6
1.99	5
2.99	4
3.99	1
4.99	2

### Gamification Elements and HBT

**Figure 2:**
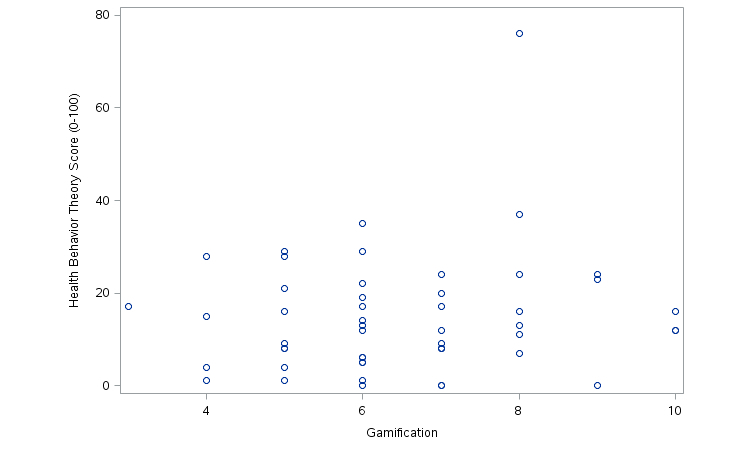
Gamification and HBT score.

The number of elements of gamification was not associated with HBT score (Spearman correlation coefficient *R*_S_=.094, *P*=.5074; Figure 2).

All of the elements of gamification were present in the sample, except for real-world prizes. The most common gamification elements in the sample were fantasy environment (96%, 50/52), whereas storyline was present in half of the sample (50%, 26/52). Rankings or standings (19%, 10/52) and leaderboards (29%, 15/50) were the least commonly utilized feature of gamification in the sample (Table 6).

**Table 6:** Gamification elements (N=52).

Gamification elements	n (%)
Storyline	26 (50)
Fantasy environment	50 (96)
Competition	31 (60)
Possibility of failure	43 (83)
Leaderboards	15 (29)
Clear expectations	49 (94)
Score	38 (73)
Ranking or standing	10 (19)
Levels	24 (46)
Real world prizes	0 (0)

## Discussion

### Principal Findings

The purpose of our study was to determine the presence of HBT in physical activity games developed for a mobile phone platform. This study also analyzed (1) the prevalence of specific health behavior constructs, (2) the association between price and presence of HBT, and (3) the association between elements of gamification and HBT in these same apps.

The presence of HBT in this sample varied and many of the apps contained low levels of HBT, but HBT levels were higher on average than HBT levels in analyses of non-game health apps. The average HBT score of this sample was 14.98 out of 100, with SuperBetter, an outlier, yielding the highest HBT score at 76. Excluding SuperBetter, the next highest HBT score was 37, and the average HBT score became 13.78. In other content analyses of non-game mobile phone apps that utilized the same coding rubric as this study, the average HBT score was lower; in a sample of physical activity apps, Cowan et al [28] reported an average HBT score of 10.01 out of 100, with a high score of 28. Similarly, West et al [30] reported in a sample of diet apps an average HBT score of 6.19 out of 100, with a high score of 26. Other content analyses of health and fitness non-game apps, including smoking cessation and weight loss apps, similarly demonstrate low levels of evidence-based health behavior change techniques [31,32,41]. There exist few content analyses of serious games for health, let alone serious health games developed for a mobile phone platform, but exergames in general may contain relatively high levels of HBT. Lyons et al [33] conducted a content analysis of physical activity video games and found significant levels of HBT elements present, such as performance feedback and modeling. Given the limited research on HBT in non-game and serious game health apps developed for mobile phones, it is difficult to determine at present whether the latter contain higher levels of HBT, though this study provides evidence to support this hypothesis.

**Figure 3:**
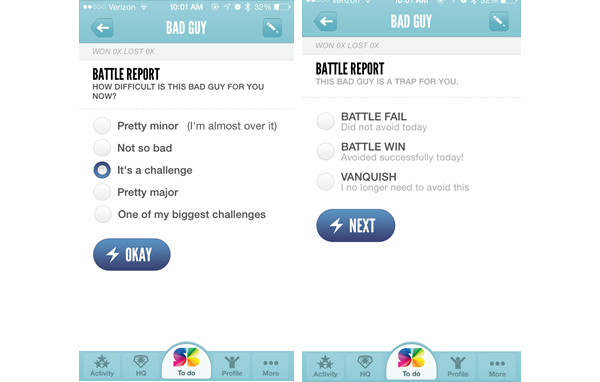
SuperBetter.

SuperBetter, an app used for achieving nonspecific health goals, was an outlier with a much higher HBT score than the sample average. SuperBetter was unique in its heavy inclusion of educational elements, including individually tailored assistance; it required feedback on not only whether users completed each exercise, but also how well users completed each exercise, as well as tips for improvement [42] (Figure 3).

**Figure 4:**
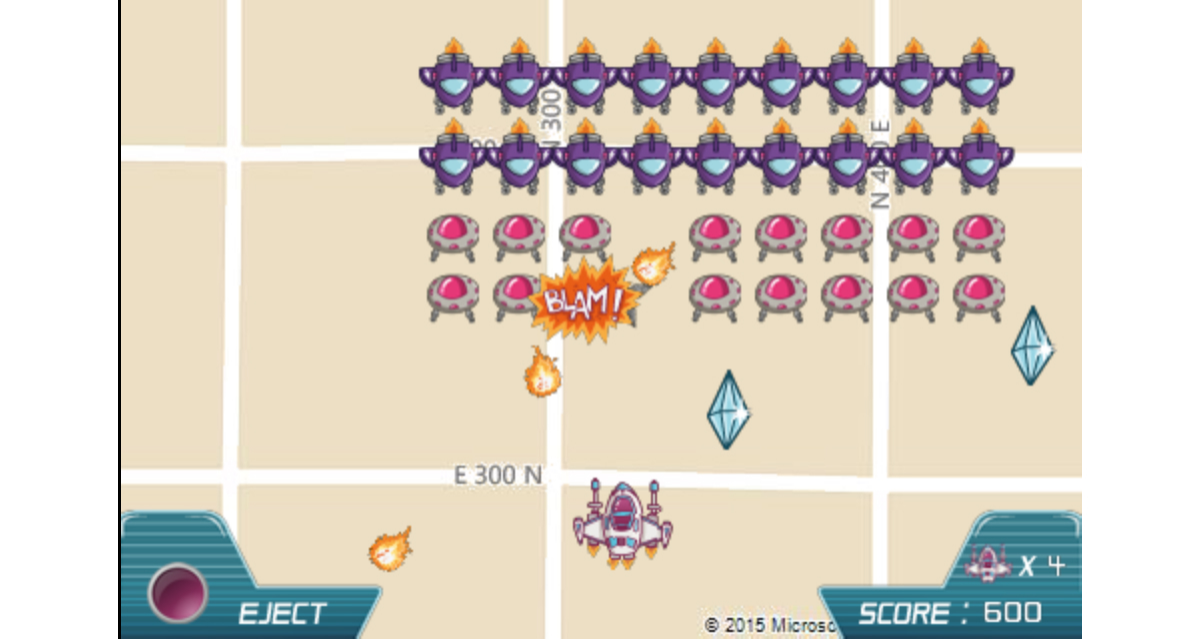
GPS Invaders.

SuperBetter stands in stark contrast to physical activity app games focused more on entertainment with few educational elements, such as GPS Invaders (Figure 4).

Despite the differences in content, both SuperBetter and GPS Invaders are considered serious games as determined by the inclusion criteria of this study. It should also be noted that the coding rubric and inclusion criteria utilized in this study emphasized the importance of educational content, in conjunction with the definition of serious games as primarily intended to educate, rather than entertain [1,19]; other definitions of serious games exist that place even less of an emphasis on educational elements [43]. It is unclear whether serious games for health require high levels of education in order to be efficacious. While games like SuperBetter contain higher levels of educational content and HBT and research suggests that interventions based in theory are more likely to lead to lasting behavior change, it is worth considering whether entertainment-based games such as GPS Invaders [44] could be more effective in changing behavior than educational games, as they may be more popular and engaging in the long-term. The interplay between educational and entertainment elements is an important area of study for serious game researchers [45]. There must be a balance between educational and entertainment elements in games to maximize player motivation and engagement [46]. Further research on the relative importance of educational versus entertainment elements in serious games for health in long-term behavior change should be conducted.

The most prevalent health behavior constructs (after gamification elements) included goal setting, self-monitoring, and self-reward. In a review of mobile apps utilized in health interventions, Payne et al [47] found that self-monitoring was the most commonly utilized health behavior construct, followed by cues to action and feedback. The use of self-monitoring in physical activity interventions has been found to be one of the strongest predictors of success in behavior change [48], and goal-setting is commonly utilized and shows promise in physical activity and obesity interventions [49]. Lyons et al [33] also found that some of the most common HBT elements in physical activity video games included feedback, modeling, rewards, and self-monitoring. While many of the apps in this sample scored fairly high in HBT and contained many HBT components shown to be effective in health behavior change, it is unclear whether the app designers consciously designed the physical activity games with behavioral theories in mind. Payne et al [47] noted in a sample of apps utilized in physical activity and diet interventions that almost all (93%) were designed with some preconceived behavioral theory or construct. However, despite the relatively high levels of HBT in this sample, it is likely these apps were not designed around HBT, especially as it appeared the majority were not designed for formal health interventions. Additionally, as suggested by Cowan et al [28], app developers come from varied backgrounds and may not have formal training in HBT. It may be that app-based serious games lend themselves well to inclusion of HBT by virtue of design, whether it is consciously included or not.

Paid apps were no more likely to include elements of HBT than free apps. However, the sample of paid apps was small and these results should be interpreted with this limitation in mind. West et al [31] and Cowan et al [28] conducted a similar content analysis of paid health and fitness apps and reported that apps exceeding US $0.99 in price were more likely to contain elements of behavioral theory [28,31]. Similarly, Abroms et al [41] found in a content analysis of tobacco cessation apps that paid apps were more likely to include evidence-based practices for tobacco cessation, suggesting that there may be a relationship between quality and price for apps. While there was no overall association between price and HBT in this study, SuperBetter, which had the highest HBT score, was also the most expensive app. It is important for consumers and health professionals to avoid assuming that paid fitness games associated with a well-known or popular organizations are more efficacious. Affiliation with a professional organization does not always imply validity. Given the wide variance in expertise of app developers, research and evaluation of health apps by both industries and independent researchers are important to determine how to design apps that will change health behaviors in the long-term. Furthermore, health app designers would do well to partner with health behavior experts.

There was also no significant association between elements of gamification and presence of other HBT constructs—that is, having more game elements did not increase the overall HBT score. Researchers disagree on definitions of serious games, and the number and type of gamification elements needed to merit the classification of a serious game vary [50]. Many legitimate serious games may not contain high levels of traditional game-like elements. Current research indicates that available health and fitness apps contain significant amounts of gamification elements [15], but these same apps do not appear to contain high levels of HBT [28,30,31]. There appears to be a difference in HBT content between physical activity games apps and non-game apps, but it seems this relationship is not linear (it is not the case that the more gamified the app, the more behavioral theory components it contains). Assuming it is true that serious health game apps are naturally better suited for HBT than nongame apps, it is unclear how many elements of gamification a game must have, or which specific gamification elements are required, for this relationship to hold true.

The findings of our study are significant for practical use in public health, especially as mobile apps are being increasingly utilized in health interventions [47]. While some researchers argue that current health behavior models are flawed [51], historically, designing theory-based public health interventions has been widely accepted as essential for lasting health behavior change [23]. Similarly, researchers indicate that mobile phone health interventions that incorporate health behavior elements are more efficacious than non-theory based interventions [51-53]. Riley et al argues that it is essential to utilize health behavior models in even the simplest of mobile phone interventions, such as a text message reminder intervention to increase attendance to medical appointments. Although this intervention incorporates built-in cues to action, if theoretical constructs such as perceived benefit of and barriers to attending appointments are not also addressed, the app will likely not be enough to prompt behavior changes [51]. It could be argued that mobile phone games—far more complex than text message interventions—may also require a theoretically based orientation for effectiveness in changing health behavior, though as mentioned previously, the dual nature of serious games as both educational and entertainment tools may complicate this relationship.

### Limitations

The findings of our study should be interpreted in the context of some limitations. First, the coders only used the mobile phone for either 1 level or 30 minutes to code for HBT elements. It is possible that some HBT components were missed by limiting use to this time frame, though unlikely; a recent study demonstrates that while mobile phone and app use is increasing, average app session length has stayed constant at about 5.7 minutes [54]. The coders spent over five times that amount coding each app, so the chances of missing unique or important HBT components appear low. Additionally, the final sample size was small, though this was difficult to avoid, as health app games are still a recent development. The coders conducted a thorough search, including the first 500 apps for every search term, to capture as many existing physical activity games as possible. While the sample could have been expanded upon to include other app games related to health (e.g., diet, nutrition), exclusively physical activity apps were chosen because previous content analyses of health apps have been similarly restrictive in scope [28,30], and addressing only physical activity games was more conducive to direct comparison between both these previous content analyses and studies of exergames.

The coders analyzed only app descriptions to determine if each app qualified as a health game, and it is possible that some games were missed due to inadequate descriptions. The coders attempted to compensate for this limitation by selecting a sample (10 apps) that appeared in the search but did not appear to meet the description of a game via the description; the coders found that none of these games fit the definition of a serious game, so the likelihood that apps were overlooked due to weaknesses in the description appears low. Finally, it should be noted that the definition of a serious game is not consistent across research; a number of legitimate serious exercise games exist that do not fulfill the criteria we proposed. Legitimate exergames (according to other researchers) may have been excluded from our sample. In this particular study, we were more interested in games emphasizing education, though content analyses of serious games with different criteria would be interesting for future research.

### Conclusions

Physical activity health games developed for mobile phones are a potentially viable option for health interventions, though further research and development of such games should continue. Further research should be conducted to determine whether these health games are efficacious in health interventions, and the extent to which educational and gamification elements impact efficacy should be further assessed as well. Collaboration between app designers and behavioral specialists is also crucial to help promote lasting behavior change. Investigations into whether serious app games for health are more conducive to inclusion of HBT and whether they universally contain more elements of HBT is valuable in order to assess whether such games can improve individual and community health in the long-term.
